# Expression analysis of *Cell wall invertase* under abiotic stress conditions influencing specialized metabolism in *Catharanthus roseus*

**DOI:** 10.1038/s41598-018-33415-w

**Published:** 2018-10-10

**Authors:** M. J. Nishanth, S. A. Sheshadri, Sudarshan Singh Rathore, S. Srinidhi, Bindu Simon

**Affiliations:** 1 Phytoengineering Lab, School of Chemical and Biotechnology, SASTRA Deemed to be University, Thanjavur, Tamil Nadu India; 2 Actinomycetes Bioprospecting Lab, School of Chemical and Biotechnology, SASTRA Deemed to be University, Thanjavur, Tamil Nadu India

## Abstract

*Catharanthus roseus* is a commercial source for anti-cancer terpenoid indole alkaloids (TIAs: vincristine and vinblastine). Inherent levels of these TIAs are very low, hence research studies need to focus on enhancing their levels *in planta*. Since primary metabolism provides precursors for specialized-metabolism, elevating the former can achieve higher amounts of the latter. Cell Wall Invertase (CWIN), a key enzyme in sucrose-metabolism catalyses the breakdown of sucrose into glucose and fructose, which serve as carbon-skeleton for specialized-metabolites. Understanding *CWIN* regulation could unravel metabolic-engineering approaches towards enhancing the levels of TIAs *in planta*. Our study is the first to characterize *CWIN* at gene-expression level in the medicinal plant, *C*. *roseus*. The *CWINs* and their inter-relationship with sucrose and TIA metabolism was studied at gene and metabolite levels. It was found that sucrose-supplementation to *C*. *roseus* leaves significantly elevated the monomeric TIAs (vindoline, catharanthine) and their corresponding genes. This was further confirmed in cross-species, wherein *Nicotiana benthamiana* leaves transiently-overexpressing *CrCWIN2* showed significant upregulation of specialized-metabolism genes: *NbPAL2*, *Nb4CL*, *NbCHS*, *NbF3H*, *NbANS*, *NbHCT* and *NbG10H*. The specialized metabolites- cinnamic acid, coumarin, and fisetin were significantly upregulated. Thus, the present study provides a valuable insight into metabolic-engineering approaches towards augmenting the levels of therapeutic TIAs.

## Introduction

Cell Wall Invertase (CWIN, EC: 3.2.1.26), a key enzyme in sucrose-metabolism catalyses the irreversible breakdown of sucrose into glucose and fructose. In addition, it also has several pleiotropic roles such as stress-response, sugar-signalling, flower, fruit and seed development^1–3^. Besides, CWIN was also found to modulate specialized-metabolites levels *in planta*^4^. Introduction of yeast *CWIN* into *Nicotiana tabacum* upregulated the levels of phenylpropanoids^5^. Plant specialized-metabolites had been thought to be of little significance, but advancements in research have unravelled their physiological and therapeutic importance^6^. Nearly 50,000 therapeutic specialized-metabolites have been identified in plants and characterized to date^6,7^.

*Catharanthus roseus* (*C*. *roseus*) is a widely-known medicinal plant, used as the predominant source of the pharmaceutically-important specialized-metabolites, especially Terpenoid Indole Alkaloids (TIAs; vincristine and vinblastine are used in anticancer treatment^8^). Dimerization of vindoline and catharanthine produces vinblastine *in planta*, which is further converted to vincristine^9^. The monomeric precursors, vindoline and catharanthine are spatially separated in the plants (vindoline is localized in laticifers and idioblasts whereas catharanthine is secreted to the leaf surface and accumulates in the wax-exudates)^10,11^. Hence the production of vincristine and vinblastine is limited to trace amounts *in planta*^10^. Owing to the inherent low-yields of the anti-cancerous TIAs, various biochemical and molecular studies have been conducted to unravel the specialized-metabolism and enhance TIA concentrations in *C*. *roseus*^12–14^. Industrial production of vincristine and vinblastine is achieved by chemical coupling of more abundant^15^ monomeric precursors-vindoline and catharanthine^16^. Therefore, increasing the yields of these precursors *in planta*, can be a plausible approach to obtain higher yields of the drugs *via* coupling process.

Most studies have shown that primary and specialized-metabolisms are intimately interconnected, the former providing the precursors to the latter^17–19^, but to date only a few attempts have been made towards understanding this interconnection, especially at the molecular level^20^. Sucrose and its hexose products (glucose and fructose) play important roles in both primary and specialized-metabolism. Besides acting as signalling molecules, they also provide carbon skeletons towards the production of specialized-metabolites^21^. The cross-talk between carbon and specialized-metabolisms has also been reported in glandular trichomes of tomato, wherein the energy and reducing power from photosynthesis are diverted towards specialized-metabolism, achieving high metabolic productivity^22^. Understanding the interplay between primary and specialized-metabolisms at molecular level involving the important genes and enzymes could unravel novel ways to enable manipulation of specialized-metabolites biosynthesis *in planta*. Despite the significant role of CWIN in primary and specialized-metabolism, as to our knowledge, so far no work has been done to understand *CWIN* regulation in any medicinal plants, including *C*. *roseus*.

As a part of our study, *CWIN* genes were identified in *C*. *roseus* genome and subjected to *in-silico* characterization, followed by tissue-specific expression analysis in the leaf, stem and roots. To understand the interrelationship between *CWIN* and major specialized-metabolism genes in *C*. *roseus*, a comparative expression analysis was performed for *CWIN* and other sucrose-metabolism genes (*Sucrose Synthase*, *SUSY; Sucrose Phosphate Synthase*, *SPS*), *TIA* pathway genes (*Geraniol-10-Hydroxylase*, *G10H; Deacetylvindoline-4-O-acetyltransferase*, *DAT; Secologanin synthase*, *SLS; Peroxidase*, *PRX1; 1-deoxyxylulose 5-phosphate synthase*, *DXS; Tryptophan Decarboxylase*, *TDC; Strictosidine synthase*, *STR*), antioxidants and senescence-associated genes (*Catalase*, *CAT; Superoxide dismutase*, *SOD* and *Senescence-associated gene*, *SAG*) under different abiotic stress conditions. The gene-expression results were further supported by metabolite analysis (includes monomeric TIAs: vindoline, catharanthine; bis-indole TIA: vinblastine). Finally, to study the effect of *C*. *roseus CWIN* on specialized-metabolism in cross-species, the full-length *CWIN* isoform (*CrCWIN2*) was transiently overexpressed in *N*. *benthamiana* leaves, followed by gene-expression and metabolite analyses.

## Results and Discussion

### Identification and *in-silico* analysis of *CWIN* isoforms from *C*. *roseus*

**Figure 1:**
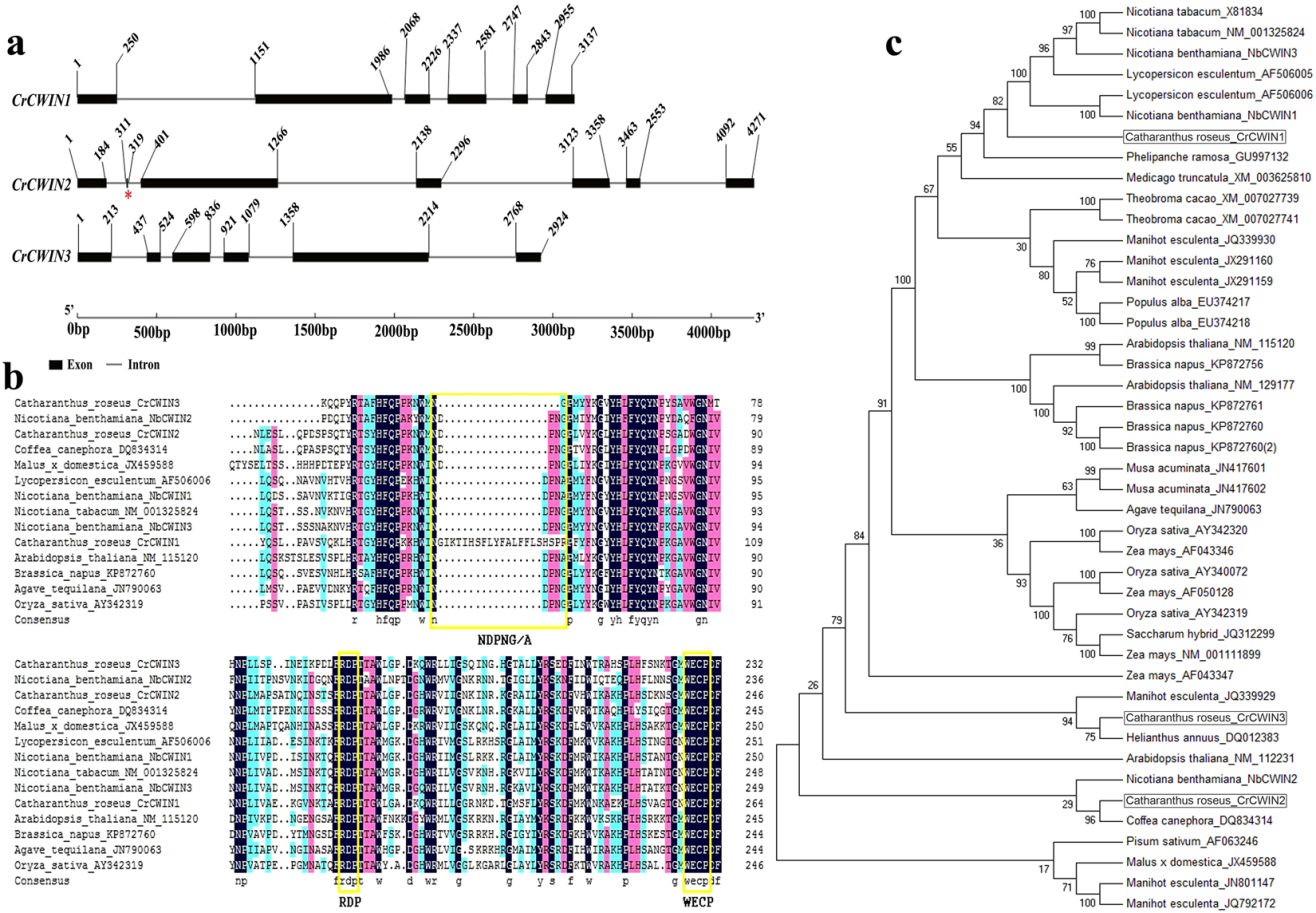
(**a**) Genomic architecture of *Cell Wall Invertase* isoforms in *Catharanthus roseus* Solid rectangles represent the exons and the lines represent the introns. The first exon starts with the start codon and the last exon ends with the stop codon. The red asterisk highlights the presence of the second mini exon (9 bp long) in *CrCWIN2*. Lengths of exons and introns of *CrCWIN* genes are displayed proportionally as indicated by the scale at the bottom. (**b**) Multiple alignment of Cell Wall Invertase amino acid sequences from various plants. The important catalytic sites of Cell Wall Invertases, ‘NDPNG’, ‘WECP’ and ‘RDP’ are highlighted in yellow. CrCWIN1 and CrCWIN3 lack the Sucrose-binding box ‘NDPNG’. WECP and RDP are conserved across the isoforms (**c**). The unrooted phylogenetic tree depecting the evolutionary relationship among CWIN isoforms of *C*. *roseus* and other plants. Names and the respective accession id’s. are indicated. Maximum likelihood method was used to construct the tree with 1000 bootstrap replicates using MEGA7 software the *C*. *roseus CWIN* isoforms are highlighted with asterisks.

Homology based analysis of the *C*. *roseus* coding sequences revealed the presence of three *CWIN* isoforms; CRO_T000083 (*CrCWIN1)*, CRO_T031716 *(CrCWIN2)*, and CRO_T020329 (*CrCWIN3)*. *CrCWIN2* (CDS length: 1725bp; Genomic scaffold: cro_scaffold_3060381) had 7 exons and 6 introns whereas *CrCWIN1* (CDS length: 1797bp; Genomic scaffold: cro_scaffold_3070386) and *CrCWIN3* (CDS length: 1713bp; Genomic scaffold: cro_scaffold_3065222) were found to have 6 exons and 5 introns each. Previously characterized invertases from *Agave tequilana*^23^, *Populus trichocarpa*^24^, Sugarcane^25^ and Cassava^26^ have been shown to contain 6–8 exons. The genomic architecture of *CrCWIN* isoforms has been depicted in Fig. 1a.

The deduced amino acid sequences of *CrCWIN1*, *CrCWIN2* and *CrCWIN3* were predicted to contain 598 (67.8 kDa), 574 (65.0 kDa), 570 (64.9 kDa) amino acid residues. All the isoforms were predicted to localize in the cell wall. These findings have been summarized in Table 1. It is known that *CWIN* from Sugarcane, *SoCIN1* encodes a protein 577 amino acids in length^25^ and *Arabidopsis thaliana CWIN* with seven exons and six introns encodes 584 amino acids with mass 66.280kDa^27^, thus highlighting the molecular similarities among *CWINs* from *C*. *roseus* and other plants.

**Table 1:** Cell Wall Invertases in *Catharanthus roseus*.

Gene Name (Sequence ID)	Scaffold	Matching sequence details (Genbank ID)	% Identity	Query coverage in tBLASTx	Length of coding sequence (in bp) (Position in scaffold)	Length of genomic gene (in bp)	Predicted amino acid length (Molecular weight; kDa)	Predicted sub-cellular localization
*CrCWIN1* (CRO_T000083)	cro_scaffold_3070386	*Nicotiana tabacum* beta-fructofuranosidase, (XM_016633086)	77%	95%	1797 (3502 to 6638)	3137	598 (67.8)	Cell wall
*CrCWIN2* (CRO_T031716	cro_scaffold_3060381	*Coffea canephora* cell-wall invertase (DQ834314)	78%	90%	1725 (12947 to 17217)	4271	574 (65.0)	Cell wall
*CrCWIN3* (CRO_T020329)	cro_scaffold_3065222	*Chicorium intybus* mRNA for putative invertase (Y11124)	61%	90%	1713 (27037 to 24482)	2924	570 (64.9)	Cell wall

*CrCWIN1* and *CrCWIN3* lack the ‘mini-exon’, generally present as the 9 bp long second exon in all the functional *CWINs*^23,28^. This exon encodes ‘DPN’, the tripeptide core of the beta-fructofuranosidase motif, ‘NDPNG’ (sucrose-binding box, directly involved in the catalysis of sucrose-cleavage^23^). Such “defective invertases” lacking the NDPNG motif are thought to be ubiquitous in plant kingdom and are commonly found in tobacco, rice, maize, potato, poplar and chicory. They are known to possess regulatory functions during pollen development^29^. The other two important catalytic sites, ‘WECP’ and ‘RDP’^28^ were present in all the three isoforms (Fig. 1b). The Cys-residue of ‘WECP’, is a conserved feature of CWINs^28^.

The evolutionary relationship among CWINs of *C*. *roseus* and other plant species was analysed *via* phylogenetic analysis (MEGA7). *Cr*CWIN1 grouped with CWINs of *L*. *esculentum* and *N*. *tabacum*; *Cr*CWIN2 was found to be closely related to *C*. *canephora* CWIN whereas *Cr*CWIN3 was found to group with CWIN of *H*. *annuus*, with well supported bootstrap values (Fig. 1c).

### Tissue specific expression profiling of *C*. *roseus CWIN* isoforms

**Figure 2:**
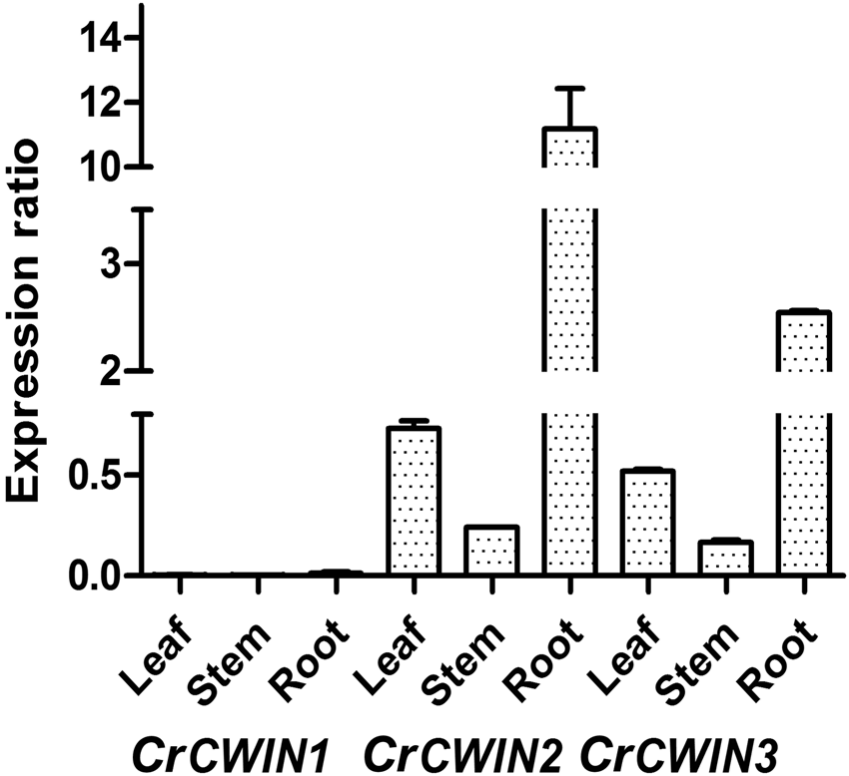
Tissue- specific expression pattern of the three *CWIN* isoforms in leaf, root and stem tissues of three month old *C*. *roseus* plants. The relative expression levels of *CWIN* isoforms in leaf, root and stem tissues were normalized against transcript levels of *SAND*. Results are represented as mean relative transcript levels and the error bars indicate standard deviation of triplicate samples.

The expression pattern of *CrCWINs* was analysed in leaf, stem and roots *via* qRT-PCR followed by LinReg PCR analysis. *SAND* was used as the internal reference gene^30^. The result, as shown in Fig. 2 depicts the mean relative expression levels of each isoform in these tissues. Overall, *CrCWIN2* (the isoform containing all the catalytic sites) showed the highest expression, followed by *CrCWIN3* and *CrCWIN1*. Highest transcript levels of *CrCWIN2* was seen in root tissues (mean relative expression ratio: 11.18), followed by leaves (0.73) and stem (0.24). *CrCWIN3* was found to have a similar trend wherein its highest expression was seen in roots (2.54), followed by leaves (0.51) and stem (0.166). High demand for hexoses in roots (sink tissues)^28,31^ is a plausible reason for the high transcript levels. A similar trend was seen in carrot, wherein the acid invertase activity correlated with the utilization and storage of sugars in sink organs^28^. In comparison to other two isoforms, the expression of *CrCWIN1* was found to be very minimal. Similar tissue-specific differential expression was also observed among maize *CWINs* wherein *Incw3* showed varied expression while *Incw4* was constitutively expressed^32^.

### Stress mediated gene expression profiling in *C*. *roseus*

TIA metabolism; specifically vindoline and catharanthine biosynthesis is known to be influenced by abiotic stresses^13^. Extensive research has been done towards understanding transcriptional responses of TIA biosynthesis genes under conditions influencing alkaloid metabolism^33,34^. Multiple RNA-Seq experiments have been conducted to understand transcriptomic-modulations under various conditions^35–42^. Gongora-Castillo *et al*.^36^ generated *C*. *roseus* transcriptome sequence and expression profiles, wherein it was found that vinblastine biosynthesis genes were up-regulated in response to methyl jasmonate treatment. Sun *et al*.^41^ investigated the transcriptional responses to *Anthranilate Synthase* (*AS*) overexpression in transgenic *C*. *roseus* hairy roots. It was found that aromatic amino acid, fatty acid, glutathione and alpha-linolenic acid metabolism-genes were significantly up-regulated, whereas glycolysis/gluconeogenesis, amino and nucleotide sugar, starch-sucrose, cysteine-methionine and pyruvate-metabolism genes were downregulated, indicating the possible modulations in primary and specialized metabolic pathways due to *AS* overexpression. Liu *et al*.^39^ studied the transcriptomic responses of *C*. *roseus* to Peanut-Witches’-broom Phytoplasma-infection *via* transcriptome sequencing. It was found that many of the abiotic and biotic stimulus- related genes as well as photosynthesis, chloroplast development and energy metabolism genes were up-regulated, indicating at the dynamic changes in primary metabolism and stress related gene-expression. Van Moerkercke *et al*.^35^ constructed CathaCyc, a metabolic pathway database of *C*. *roseus*, based on RNA-Seq data. Though gene-expression studies have been conducted in *C*. *roseus*, the correlation between the expression patterns of *CWINs* and TIA biosynthesis genes has not been investigated.

**Figure 3:**
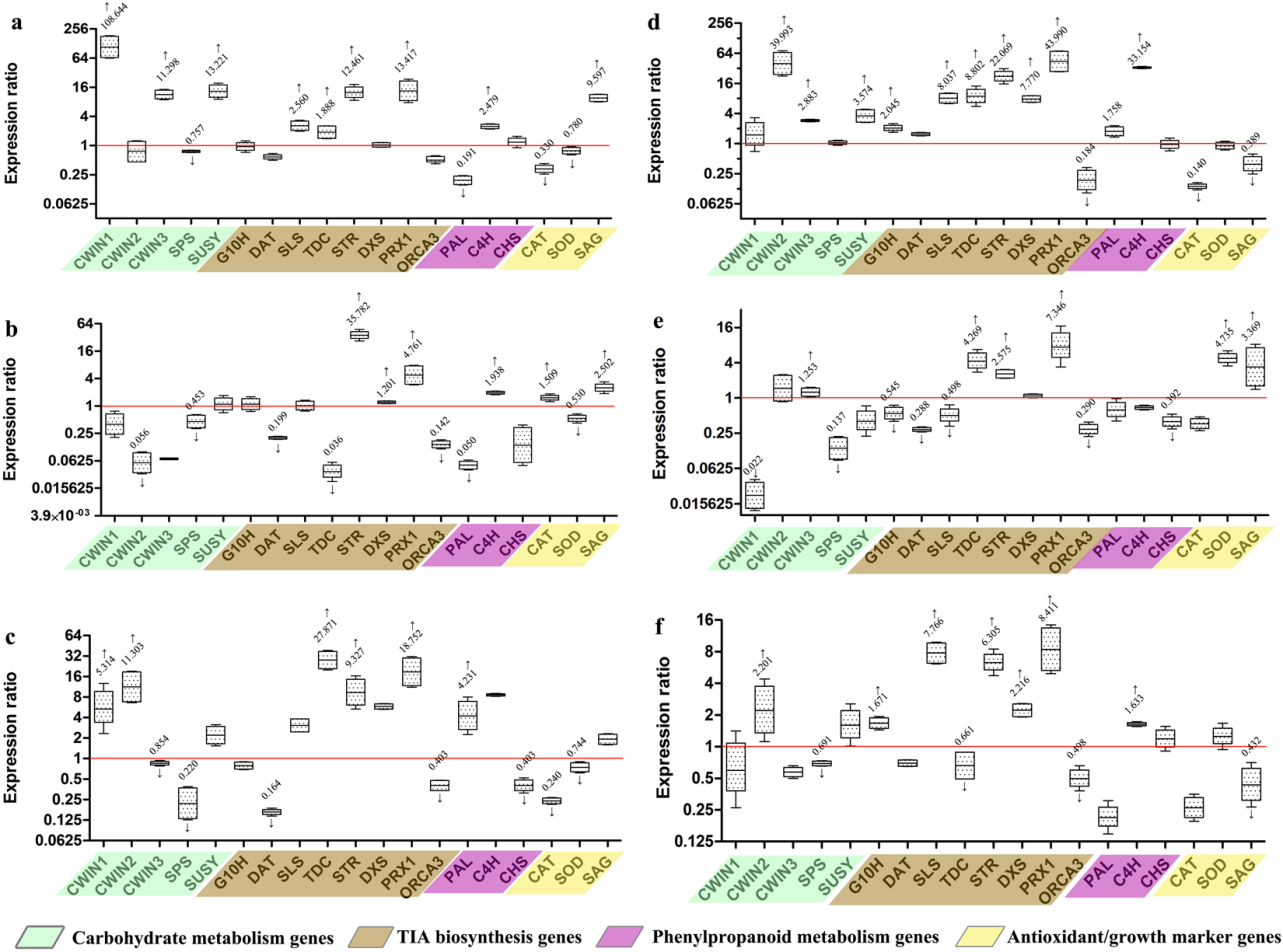
Gene expression profiles of soluble sugar, TIA metabolism genes, phenylpropanoid metabolism and antioxidant genes. Expression profile in (**a**) cold stress, (**b**) drought, (**c**) salinity, (**d**) sucrose, (**e**) UV radiation and (**f**) wound- treated tissues. The Results depict statistically significant (p < 0.05) Up/downregulation of the considered genes determined *via* three independent replicates in qRT-PCR. Data were analysed using LinREG PCR and REST software. Mean factors of gene expression compared to control group are represented as boxplots. Corresponding expression ratios of the genes significantly affected (p < 0.05) are shown next to the whisker-boxes. The median expression ratio values above/ below 1.0 indicate up/ downregulation of the target gene under stress treatment compared to the control leaves, indicated using upward and downward arrows.

In the present study, *C*. *roseus* leaves were subjected to cold, drought, salinity, UV radiation, wounding and also exogenous sucrose treatment. The expression pattern of major genes involved in TIAs biosynthesis was monitored. Also, to study the simultaneous effect on other metabolic pathways, carbohydrate, phenylpropanoid metabolism and antioxidants/growth-associated genes were analysed. All the expression ratios have been depicted in Fig. 3 and the statistically significant (P < 0.05) results are detailed below.

#### Cold stress

As shown in Fig. 3a, cold stress resulted in the upregulation of sucrose-metabolizing genes (*CrCWIN1*, *CrCWIN3* and *SUSY*), whereas *SPS* was downregulated. Sugars such as glucose, fructose, sucrose, raffinose and stachyose^43^ are well-known cryoprotectants, mainly involved in protecting cell-membrane integrity by reducing freeze-induced dehydration^44,45^. Cold-responsive upregulation of *SUSY* and *CWIN* isoforms has been previously documented^46^. While previous studies have shown an upregulation of *SPS* under cold stress^44,46^ our results showed a slight decrease in its expression, probably owing to species-specific differences. In response to low temperature stress, plants modulate the expression of genes involved in soluble sugar metabolism and transport, and also starch breakdown^45,46^, thereby accumulating sugars including sucrose and hexoses that act as cryoprotectants. Thus, a cascade of sugar metabolism genes, transporters and signalling components (such as kinases) is involved in cold stress-response *in planta*. The differential expression patterns of these genes vary in a species-specific manner^47^. As for the TIA metabolism genes, *SLS*, *TDC*, *STR* and *PRX1* were induced significantly, indicating at a possible role of TIAs in cold stress response. Peroxidases are known to impart cold-tolerance^48^. A differential expression pattern was observed for the phenylpropanoid-biosynthesis genes. Phenylpropanoids, specifically flavonoids impart freeze tolerance by preventing protein aggregation^49^. A similar report in *A*. *thaliana* presented a slightly differing pattern, wherein *PAL* was found to be upregulated along with most of the carbohydrate metabolism genes^50^. Interestingly, the antioxidant gene *CAT* and *SOD* were downregulated while *SAG* was upregulated, indicating that *PRX1* may have a more dominant anti-oxidant role compared to *CAT* and *SOD*.

#### Drought stress

Drought stress was found to have adverse effects wherein most of the tested genes were downregulated (Fig. 3b). The carbohydrate metabolism genes were mostly downregulated. Drought inhibits plant growth, disturbs mineral-nutrient relations and impairs metabolism due to changes in photosynthetic carbon metabolism^51,52^. While two of the TIA-biosynthesis genes (*DAT* and *TDC*) were repressed, significantly high upregulation was observed for *STR* and *PRX1*, probably attributed to the increased demand of turgor pressure^53^. The phenylpropanoid biosynthesis genes again showed a differential expression pattern. Elevated levels of phenolics and their biosynthesis genes is a characteristic of drought-stressed tissues^53^. Cell wall toughening during drought was associated with enhanced lignin (a derivative of phenylpropanoid pathway) biosynthesis^53–55^. The antioxidant genes showed a differing trend, while *SAG* was elevated under drought. These observations are in agreement with previous report wherein it was found that drought stress causes modulations in C/N ratio, decreased growth and senescence onset in sorghum^56^.

#### Salinity

As shown in Fig. 3c, salinity had varying effects on the expression of sucrose-metabolism genes, wherein *CrCWIN1* and *CrCWIN2* were highly induced, while *CrCWIN3* and *SPS* were repressed. Osmoregulation is a key aspect in salinity tolerance in plants and some of the major osmolytes like proline, sugars and polyols play pivotal role in alleviating salt stress, thus explaining the elevated levels of sugar metabolism genes^57^. Among the TIA metabolism genes, *TDC*, *STR* and *PRX1* were upregulated, while *DAT* was downregulated. Alkaloids are known to impart salinity tolerance to plants further corroborating their role in alleviating salinity stress^58,59^. Our results indicating the elevated levels of *CWINs*, *STR*, *TDC* and *PRX1* under salinity therefore provide a prospective molecular-crosstalk between carbohydrate and TIA-biosynthesis pathways. The phenylpropanoid gene *PAL* was induced, while *CHS* was repressed. Similar reports have indicated that salt treatment could upregulate phenylpropanoid biosynthesis genes in safflower^60^, *Salvia* species^61^ and *Caragana korshinskii*^62^.

#### Sucrose-supplementation

Exogenous sucrose treatment had a marked effect on the soluble sugar, phenylpropanoid and TIA metabolism genes, wherein the expression of most of the genes examined was found to be upregulated (Fig. 3d). Sucrose treatment resulted in a highly pronounced upregulation of *CrCWIN2*, but not of *CrCWIN1* or *CrCWIN3*, possibly due to the lack of the sucrose-binding box in these two isoforms. Further, *SUSY* was also found to be upregulated. The TIA-biosynthesis genes, *G10H*, *SLS*, *TDC*, *STR*, *DXS* and *PRX1* were found to be significantly induced. Among the phenylpropanoid genes, *PAL* and *C4H* were considerably upregulated. It has been suggested that sucrose-supplementation induces *CWIN* in potato, along with principal phenylpropanoid genes, caused by a network of transcription factors (*WD40*, *AN1* and *bHLH*^63^). Sucrose-supplementation has been known to improve the therapeutic TIAs and phenylpropanoids^64^. Further, *CAT* and *SAG* were repressed, indicating a possible reduction in oxidative stress and senescence.

#### UV stress

As shown in Fig. 3e, UV treatment downregulated *CrCWIN1*, while TIA metabolism genes showed a varying trend. *G10H*, *DAT* and *SLS* were downregulated, while *TDC*, *STR* and *PRX1* were upregulated. Previous reports also found an upregulation in the expression of *STR* and *TDC*^65^. UV-mediated alkaloid enhancement is attributed to their UV-absorbing properties, which prevents the damage to photosystems caused by UV-B radiation^45,66^. Further, *CHS* was considerably downregulated, which is in agreement with the previous observation in *A*. *thaliana* wherein several phenylpropanoid metabolism genes were downregulated in plants exposed to UV radiation^67^. However, the sensitivity of plants to UV radiation has been shown to vary with different plant species^68^. The antioxidant and senescence marker genes *SOD* and *SAG* were upregulated, possibly indicating elevated demand for ROS scavenging mechanisms due to UV stress.

#### Wounding stress

As depicted in Fig. 3f, wounding stress resulted in a slight upregulation of *CrCWIN2*, while repressing *SPS*, indicating a possible enhancement in sucrose breakdown and reduction in its synthesis. Remarkably, sugars are known to regulate the expression of wound-inducible genes, such as pathogenesis-related genes^69^, thereby corroborating the influence of wounding on soluble sugar metabolism genes. TIA-biosynthesis genes (*SLS*, *STR*, *DXS* and *PRX1*) were largely upregulated. In *C*. *roseus*, wounding is known to activate MAP-K mediated signalling cascade and subsequently, the genes and regulators of TIA-biosynthesis pathway^33^. Our results further indicated that except *C4H*, all the phenylpropanoid biosynthesis genes were repressed. This observation showed that resource allocation might be directed towards lignin biosynthesis in response to wounding.

TIA metabolism in *C*. *roseus* is under tight regulation at transcriptional level by several transcription factors such as *ORCAs*, *CrBPF1*, *CrWRKY1*, *CrMYC1*, *CrMYC2*, *BIS1*, *GBF2* and *ZCTs*^70^. *Cr*WRKY1 binds to the promoter of *TDC* and its overexpression resulted in the upregulation of several genes, especially regulating the serpentine pathway. *Cr*BPF1 (a MYB transcription factor) is known to repress TIA levels. *CrGBF1*, *CrGBF2* and the Zinc Finger Transcription factors- ZCT-1, 2, 3 are known transcriptional repressors of TIA biosynthesis. ORCA3, an AP2/ERF factor is a master regulator of primary and specialized metabolism in *C*. *roseus*^71^ and is known to play critical role in TIA biosynthesis^70^. ORCA3 transactivates the expression of *Strictosidine synthase* (*STR*), a key TIA biosynthesis gene by binding to its 5′ upstream-*cis*-element, jasmonate and elicitor-responsive element (*JERE*)^71^. Further, it upregulates the expression of several structural genes such as *TDC*, *D4H*, *SLS*, *CPR*, *DXS* and *AS*)^71^. Considering the importance of *ORCA3* in TIA biosynthesis, its expression profile was monitored in our study. Remarkably, *ORCA3* was found to be downregulated in sucrose, UV, salt and drought treated *C*. *roseus* leaves, while in cold and wounding, there was no significant change observed. However, the TIA-biosynthesis genes were found to be upregulated under these conditions, indicating the possibility of additional regulatory components besides *ORCA3*, operating under these conditions.

To summarize, sucrose treatment was found to simultaneously upregulate *CrCWIN2* and major specialized-metabolism genes, along with downregulation of antioxidant systems (*CAT*) and senescence marker (*SAG*), indicating a reduction in oxidative stress and senescence. This observation points at a possible pattern of co-regulation in primary and specialized-metabolism gene networks in response to sucrose feeding in *C*. *roseus* leaves.

### Metabolite analysis of stress-treated leaf tissues

**Figure 4:**
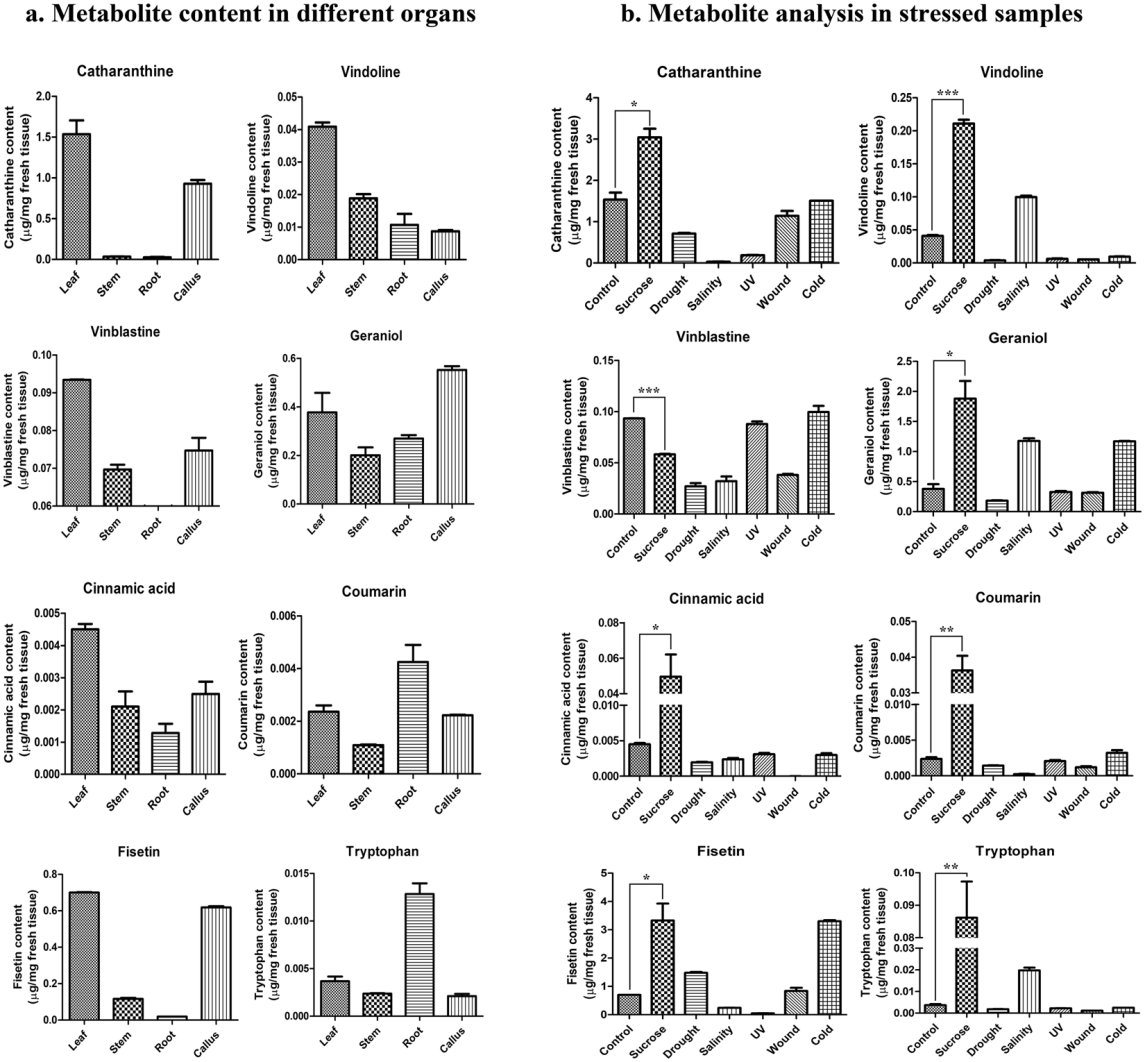
Quantification of TIAs (vindoline, catharanthine and vinblastine), specialized metabolites (geraniol, cinnamic acid, coumarin and fisetin) and tryptophan (TIA precursor amino acid) in the (**a**) leaf, stem, root and calli of *C*. *roseus* as well as (**b**) leaves subjected to different conditions influencing TIA metabolism: sucrose supplementation, drought, salinity, UV, wounding and cold stress. The error bars represent standard deviation of duplicate measurements. Statistical significance between sucrose-treated and control samples was tested using student’s t-test (*<0.05; **<0.01; ***<0.001).

Plant metabolome undergoes profound changes in response to abiotic stress^72^. In order to study the effect of stress treatments on specialized-metabolites in *C*. *roseus*, we assessed the levels of the monomeric precursors of anticancer TIA’s-vindoline and catharanthine, along with the bis-indole alkaloid-vinblastine, cinnamic acid (product of PAL catalysed reaction), coumarin, fisetin (phenylpropanoids) and geraniol (a monoterpenoid-alcohol with limiting role in TIA-biosynthesis^73^). *C*. *roseus* leaves were found to contain highest amounts of the therapeutic TIAs (Fig. 4a), therefore the leaf tissues were selected for analysing changes in metabolite amounts under the selected conditions.

It was observed that cold treatment led to a significant decrease in vindoline, while no change was observed in the levels of catharanthine. In a previous report^34^, catharanthine and vindoline accumulation was shown to be downregulated in response to cold. A precursor of TIAs, geraniol was found to be enhanced in cold-treated leaves. However, a previous report on geranium indicated that low temperature decreased geraniol levels, although the precise mechanisms are unclear^74^. Phenylpropanoids form the first line of defence against abiotic stress, owing to their inherent antioxidant potential^75^. Accumulation of cinnamic acid was found to be decreased. These results further correlate with the expression profile of phenylpropanoid genes, wherein *PAL* was found to be downregulated. However, existing reports present contrasting findings, indicating the species-specific nature of phenylpropanoid regulation under low temperature stress^76–78^. There was no change observed in the levels of coumarin. In *Arabidopsis* leaves, the levels of scopoletin, a coumarin-derivative was found to increase in response to cold treatment^79^. The differences in observations can be due to the differences in metabolic reorganization of individual plants in response to stress. Cold stress resulted in a remarkable increase in the levels of fisetin. Flavonoids are known to accumulate in response to abiotic stresses, thereby conferring tolerance to low temperatures^49,80^.

Drought stress led to a decrease in levels of all the metabolites analysed, except for fisetin. Drought has been shown to cause dynamic variations in the levels of vindoline and catharanthine, wherein vindoline displayed a decline-rise trend while catharanthine showed a gradual decline in its levels in *C*. *roseus* tissues subjected to PEG-induced drought stress^81^. Geraniol content was found to decrease upon drought treatment. The levels of geraniol in plants was shown to depend on the intensity of drought stress, duration and the species^82^. Cinnamic acid levels were found to be decreased, which correlate with our gene-expression data, wherein *PAL* was significantly downregulated. However, previous reports have shown contrasting results^83,84^. Accumulation of fisetin showed a marked increase in drought-treated tissues, indicating a possible drought-mediated upregulation of flavonoid biosynthesis. A differential effect of drought on flavonoid biosynthesis was reported in wheat^85^. Flavonoids act as ROS quenchers, thereby forming first-line of defense against oxidative stress^86^. Coumarin levels were found to be decreased. A similar result was observed in case of *Vitis vinifera* leaves, wherein most of the abundant phenolic compounds underwent a significant decline, despite other reports indicating at the converse, owing to the species-specificity of drought-induced changes in metabolite levels^87^.

Salinity stress resulted in a significant increase in the levels of vindoline, while catharanthine was severely reduced. Previous reports suggest conflicting effects of salinity on TIA levels^34,88^. Geraniol concentration increased in salt-treated tissues, thereby pointing at an upregulation of monoterpenoid biosynthesis. A similar observation was made in *Coriandrum sativum*, attributed to the increased density of oil glands^89^. Cinnamic acid concentrations showed a significant decrease in salt-treated tissues. However, a differential effect of salt stress on PAL isoforms was observed in diverse plant species^60,84,90,91^. It could therefore be inferred that the effect of salinity on cinnamic acid is species-dependent. The levels of fisetin showed a drastic decline under salinity. Research reports have indicated that pattern of flavonoid accumulation under salinity stress is species-specific, governed mainly *via* the predominant flavonoid present^92–94^.

UV stress resulted in a downregulation of TIAs and phenylpropanoids. On the contrary, previous studies have reported UV based induction of TIAs under Nitrogen-supplementation to *C*. *roseus* leaves^95^. An increase in vindoline and catharanthine levels was also reported in *C*. *roseus* suspension cell cultures subjected to UV radiation^65^. The levels of geraniol and coumarin were not affected by UV stress. UV stress has been shown to have differential effects on geraniol in different plants, attributed mainly to ROS-mediated signalling^96^. UV radiation has been proposed to induce the biosynthesis of UV-absorbing and ROS-scavenging phenols^97^ however, we report that the levels of phenylpropanoids either show a decrease (cinnamic acid and fisetin) or no change (coumarin). This could be attributed to plant-specific differences in response mechanisms to UV-exposure.

Wounding stress downregulated the production of catharanthine, vindoline, cinnamic acid and coumarin, whereas geraniol and fisetin were not significantly affected. Alkaloid formation was shown to be reduced in detached plant parts subjected to wound stress in *C*. *roseus*, attributed to the developmental-specific regulation^98^. Vázquez-Flota *et al*.^99^ reported an increase in vindoline and ajmalicine levels of wounded *C*. *roseus* seedlings, while catharanthine levels remained unaffected. Cinnamic acid esters are known to have wound-protectant effects and phenylpropanoid-derived metabolites such as acetosyringone play roles in wound stress- response^100^. The observed decline in cinnamic acid levels can be due to the channelling of this compound towards the synthesis of other downstream wound-protectant metabolites.

Exogenous sucrose-supplementation resulted in highly pronounced upregulation in the biosynthesis of all the metabolites analysed. Sucrose can act as signalling molecule inducing the biosynthesis of various specialized-metabolites such as flavonoids and anthocyanins^63,101^. Though the levels of vindoline and catharanthine were significantly increased upon sucrose treatment, the dimeric alkaloid, vinblastine was not upregulated upon any of the stress treatments. This could be attributed to the spatial separation of its precursors, vindoline and catharanthine in the leaf tissues^10,11^. Previous attempts towards enhancing TIAs in *C*. *roseus* have also shown upregulation of the monomeric precursors, rather than the dimeric TIAs. Overexpression of *ORCA3* and *G10H* had a more pronounced effect on the accumulation of the precursors (strictosidine, vindoline, catharanthine and ajmalicine) than the dimeric TIAs (anhydrovinblastine and vinblastine)^12^. Transient overexpression of *CrMPK3* also resulted in a higher upregulation of vindoline, catharanthine and serpentine compared to vincristine (dimeric TIA)^33^. Moreover, the levels of vindoline and catharanthine *in planta* are inherently higher than those of the dimers, thereby enabling researchers to commercialize their *in-vitro* coupling to obtain the dimeric TIAs^102,103^. Thus, strategies for increasing the levels of vindoline and catharanthine *via* sucrose-metabolism in *C*. *roseus*, followed by their isolation and chemical- coupling to obtain the dimeric TIAs would be promising towards enhanced production of anti-cancer TIAs.

In summary, sucrose-supplementation could enhance the production of specialized-metabolites in *C*. *roseus* leaves without causing damage to growth-associated processes. Further studies to dissect the mechanistic aspects of this effect could open up novel avenues in metabolic engineering of medicinal plants. Figure 4b summarizes the effect of the stress treatments on metabolite accumulation in *C*. *roseus*. The chromatograms (recorded at 210 nm, 250 nm and 269 nm) are available in Supplementary Fig. 1a–c.

### Isolation, cloning and transient overexpression of *CrCWIN2* CDS in *N*. *benthamiana*

**Figure 5:**
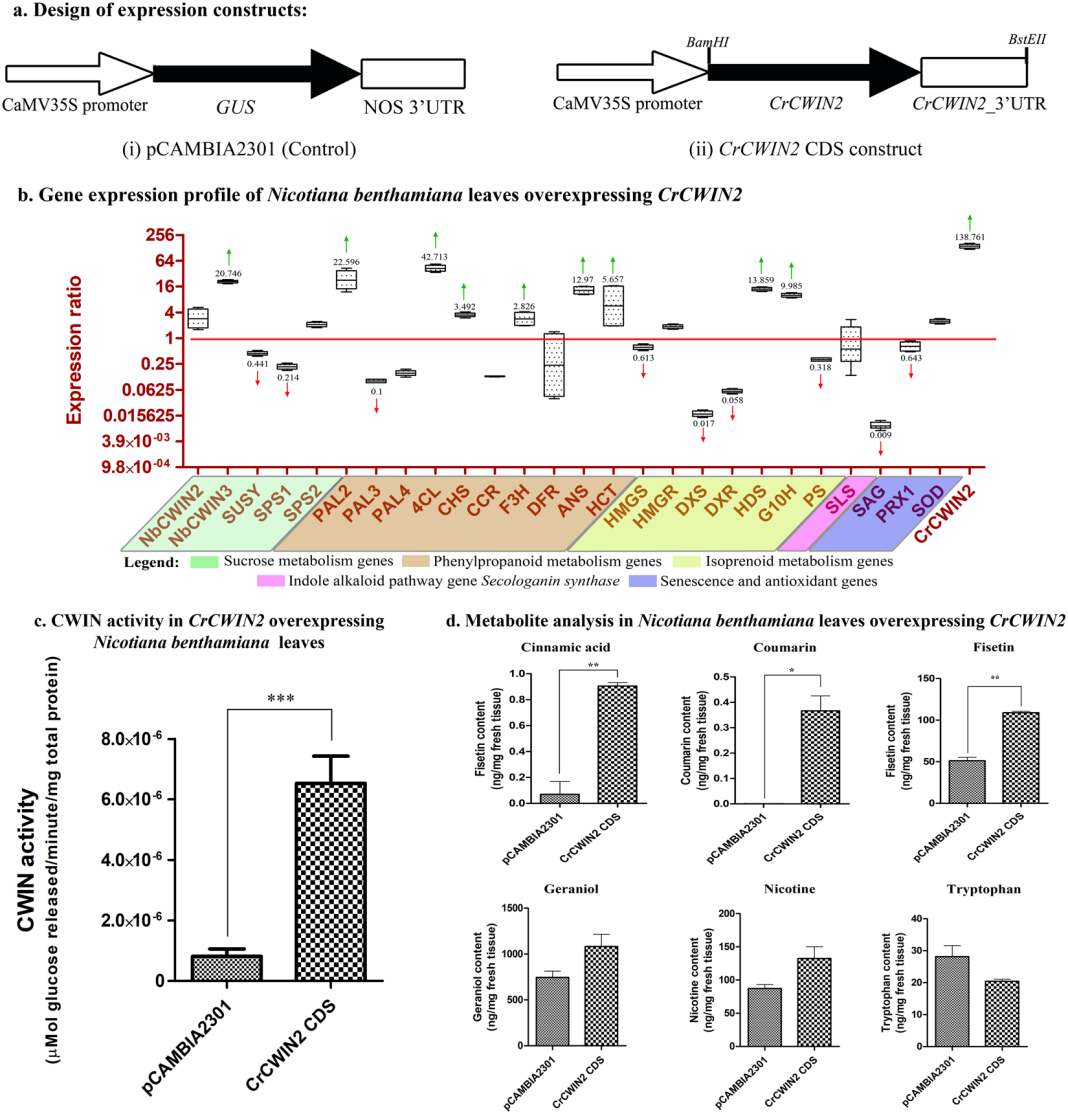
Heterologous expression of *C*. *roseus CWIN* CDS in *N*. *benthamiana*. (**a**) Expression construct carrying the [i] control vector pCAMBIA2301 and [ii] recombinant pCAMBIA2300 with *CrCWIN2* CDS downstream to CaMV35S promoter. (**b**) Expression profile of endogenous genes involved in carbohydrate metabolism, isoprenoid biosynthesis, phenylpropanoid metabolism and growth-associated genes in the *N*. *benthamiana* leaves transiently overexpressing *CrCWIN2*. Upregulation and downregulation have been indicated using an upward (green) and downward (red) arrow respectively. The numbers indicate expression ratios computed via REST^©^ software using pCAMBIA2301 vector-infiltrated sample as control. (**c**) CWIN activity in control (pCAMBIA2301) versus *CrCWIN2* infiltrated *N*. *benthamiana* leaves 4-days post infiltration. (**d**) Quantification of specialized metabolites (cinnamic acid, coumarin, fisetin and geraniol), alkaloid (nicotine) and tryptophan in the *N*. *benthamiana* leaves overexpressing *CrCWIN2* compared to pCAMBIA2301 infiltrated leaves. Mean and standard deviations (error bars) of triplicate reactions are represented. Statistical significance (P < 0.05) of the differences in means was analysed using t- test.

The overexpression of *CrCWIN2* in *N*. *benthamiana* resulted in 138-fold higher accumulation of *CrCWIN2* (Fig. 5a,b). The CWIN activity in infiltrated tissues was found to be ~2.2 times more than the control (*N*. *benthamiana* leaves infiltrated with recombinant agrobacterium carrying the vector pCAMBIA2301), thereby validating the functionality of *CrCWIN2* (Fig. 5c). The results further showed changes in the expression of endogenous genes belonging to diverse pathways. Most notably, among the sucrose-metabolism genes, *NbCWIN3* was significantly enhanced, while *NbSPS1* and *NbSUSY* were downregulated. This pattern suggested that heterologous expression of *CrCWIN2* could simultaneously alter the sucrose-synthesis as well as breakdown processes, which are respectively governed mainly *via SPS* and invertases. Among the phenylpropanoid biosynthesis genes, it was observed that *NbPAL2* was upregulated significantly, pointing at the possible role of metabolic-restructuring caused by *CWIN* overexpression. Further, the genes downstream to *PAL* revealed an interesting pattern, wherein the lignin-pathway genes (*Nb4CL* and *NbHCT*) and anthocyanin-biosynthesis genes (*F3H* and *ANS*) were also found to be significantly induced (Fig. 5b). In the isoprenoid-biosynthesis pathway, the mevalonate-biosynthesis genes (*NbHMGR*, *NbHMGS*) showed no pronounced changes in their expression, while the non-mevalonate pathway genes (*NbDXR*, *NbDXS*) were repressed. However, the downstream genes of isoprenoid biosynthesis pathway: *NbHDS*, and *NbG10H* were found to be upregulated (Fig. 5b). This observation further points at the possible interconnection between sucrose-metabolism and other specialized-metabolic processes.

The metabolite analysis of *CrCWIN2* overexpressing *N*. *benthamiana* leaves against the agroinfiltrated control showed a significant increase in the levels of specialized metabolites- cinnamic acid, coumarin, and fisetin (Fig. 5d). This indicated that *CWIN* overexpression could possibly enable partitioning of intermediates towards biosynthesis of specialized-metabolites. A previous study also reported that overexpression of yeast *CWIN* could enhance the levels of phenylpropanoids^5^ as an inherent mechanism towards protecting plant systems from pathogen-induced stress. Moreover, research evidence pointed that anthocyanins and flavonoids were recruited mostly under stressed conditions *in planta*, wherein tissue ROS content is usually higher^86^.It is also known that the metabolite influx for lignin biosynthesis occurs through sucrose *via* the shikimic acid and phenylpropanoid biosynthesis pathway^104^. The chromatograms (recorded at 210 nm, 250 nm and 280 nm) are available in Supplementary Fig. 2.

## Conclusion

Primary and specialized-metabolisms in plants are interconnected as primary metabolites can serve as precursors and signals for the synthesis of specialized-metabolites^18,19^. A clear molecular understanding of this interconnection can lead to novel metabolic engineering approaches for enhancing the biosynthesis of therapeutically important plant specialized-metabolites. *C*. *roseus*, the source of anti-cancer TIAs is an important medicinal plant in which the specialized-metabolism, especially TIA-biosynthesis has been extensively studied^14^. But, the genetic understanding of TIA-biosynthesis in relation to central carbon metabolism is lacking. A sucrose-cleaving enzyme, CWIN plays pivotal roles in modulating diverse specialized-metabolic pathways *in planta*^4^. In spite of research reports indicating at the profound effects of CWIN on specialized-metabolites biosynthesis, as to our knowledge, there have been no studies done towards understanding CWIN expression, regulation and its influence on specialized-metabolism in medicinal plants. The present work is the first to understand the possible interrelation between *CWIN* expression and TIA biosynthesis in the anti-cancer medicinal plant *C*. *roseus*.

This study identified three *CWIN* isoforms in *C*. *roseus*, which exhibited tissue-specific differential expression patterns. Among the three isoforms, only one (*CrCWIN2*) was found to possess the catalytic sites required for invertase functionality. Gene-expression analysis was performed to decipher the possible correlation between the expression patterns of *CWIN* isoforms, TIAs, phenylpropanoid biosynthesis genes, sucrose metabolism genes and also growth/ antioxidant genes under abiotic stress conditions known to influence vindoline and catharanthine production in *C*. *roseus*. Sucrose-supplementation was found to enhance the expression of *CWIN* and specialized-metabolism genes, and also improved the levels of vindoline, catharanthine, geraniol, fisetin, coumarin and cinnamic acid. The interconnection between primary and specialized-metabolism was further confirmed *via* transient overexpression of full-length *CrCWIN2* in *N*. *benthamiana*.

These results can give us cues for further metabolic engineering approaches to enhance the production of medicinally/economically important phytochemicals without compromising the overall plant health and vegetative growth. In this regard, future studies to identify the regulatory factors that can co-regulate *CWIN* and specialized-metabolism genes can be of interest.

## Materials and Methods

### Plant materials and stress treatments

Seeds of *C*. *roseus* (var. Pacifica Cherry red) were germinated on coco peat and maintained at 25 °C and 65% relative humidity in green house. Two months old *C*. *roseus* plants were subjected to different abiotic stress treatments. UV treatment was done by exposing the plants to UV radiation (48 μWcm^−2^) in LAF for two minutes^33^. Wounding was performed by damaging ~50% of the leaf lamina with a surgical blade^105^. Cold stress was induced by incubating the plants at 4 °C^105^. The detached leaves were subjected to salt stress by dipping them in 200 mM NaCl solution^105^. Drought treatment was performed by placing the detached leaves on dry blotting paper in petri dishes^105^. Exogenous sucrose treatment was performed by placing the detached leaves in 90 mM sucrose solution^101^. The tissues were harvested after 24 hours of stress treatments, snap-frozen in liquid nitrogen and stored at −80 °C.

### RNA isolation and Quantitative RT-PCR

To study the tissue-specific expression patterns of *CWIN*, total RNA was isolated from leaf, stem, root and callus tissues of *C*. *roseus*. To examine the stress mediated expression of *CWIN* along with other genes, RNA was extracted from pooled tissues of *C*. *roseus* leaves subjected to stress treatments using Plant RNA isolation kit following the manufacturer’s instructions (MN, Germany). 6 µg of total RNA was subjected to DNase treatment using RNase free DNase (Thermo Scientific, Lithuania) followed by first strand cDNA synthesis using PrimeScript RT reagent kit (TaKaRa, Japan). qRT-PCR was performed on Mastercycler Realplex qRT-PCR instrument (Eppendorf). Reaction mix contained 1 µl of diluted (3.5 times) cDNA, 5pmol each of forward and reverse primer, 1X SYBR green (Roche, Germany) in 7.5 µl reaction. Cycling parameters were: Initial denaturation at 95 °C for 5 min, 40 cycles of denaturation at 95 °C for 30 s, annealing at 52 °C for 40 s, extension at 72 °C for 30 s followed by final extension at 72 °C for 5 min.

The leaf tissues subjected to stress treatments were used for analysis of the expression levels of primary and specialized-metabolism genes using specific primers listed in Supplementary Table S1. *SAND* served as the reference gene^30^. The expression patterns of sucrose-metabolism genes: *CWIN*, *SUSY* and *SPS* along with predominant TIA metabolism genes: *G10H*, *DAT*, *SLS*, *PRX1*, *DXS*; Phenylpropanoid metabolism genes: *PAL*, *C4H*, *CHS*, *TDC*, *STR*, *CAT*, *SOD*, *SAG* and APETALA2-domain transcription factor *ORCA3*, (a known transcriptional regulator of TIA-biosynthesis genes^106^) were monitored.

Reaction efficiencies and Cq values of triplicate qRT-PCR assays were obtained through LinReg PCR software^107^. Using these values, the relative gene-expression ratios were computed *via* the Relative Expression Software Tool (REST^©^). REST^©^ performs randomization tests to determine the expression ratio of a sample, using the efficiency- corrected comparative Cq values. The up/downregulation of a gene is determined by taking into account the individual amplification efficiencies of target and reference genes^108^.

### High-Performance Liquid Chromatography (HPLC) analysis

The HPLC analysis was done as described in Singh *et al*.^102^ and Lin *et al*.^109^, with modifications. The *C*. *roseus* and *N*. *benthamiana* tissues were harvested and ground frozen in liquid nitrogen. The samples were sequentially extracted with 1:10 w/v ratio of tissue: solvent in a sequence of chloroform, followed by ethyl acetate and finally methanol each for 30 min with vigorous shaking. The supernatants were collected by centrifugation and freeze dried. The extracts were made upto 1 ml using acetonitrile and pooled together in equal ratios. Catharanthine, vindoline, vinblastine, cinnamic acid, coumarin, fisetin, geraniol and nicotine were used as standards, purchased from Chemfaces, China. The samples were filtered through a 0.22 µm syringe-driven filter and analysed *via* a reverse-phase HPLC system (Agilent 1260-Infinity, C-18 column 4.7 × 250 mm; 5 µm particle size) at 25 °C. The mobile phase consisted of 0.1% formic acid (A) and acetonitrile (B). The elution profile was as follows: 0 min: 100% A; 0–5 min: 100–70% A; 5–25 min: 70–50% A; 25–28 min: 50–30% A; 28–30 min: 30–100% A; 30–35 min: 100% A. The flow rate was maintained at 1 ml.min^−1^. The injection volume was 20 µl and the eluent was monitored using a PDA-DAD detector between 190 nm and 400 nm. The concentrations of the selected compounds were calculated by comparing the peak area, retention time (RT) and wavelength of the designated compound and expressed in µg of compound per mg of fresh tissue (*C*. *roseus*) and ng of compound per mg of fresh tissue (*N*. *benthamiana*).

### Identification and bioinformatic analysis of *C*. *roseus CWIN* coding sequences (CDS)

The amino acid sequences of the well characterized CWINs of *Arabidopsis thaliana* (AT3G13790), *Nicotiana tabacum* (X81834), *Coffea canephora* (DQ834314), *Lycopersicon esculentum* (AF506006) and *Oryza sativa* (AY342319) were used as query sequences to find the *CWIN* coding sequence isoforms in *C*. *roseus via* tBLASTn analysis of Medicinal Plant Genomics Resources (MPGR) Consortium (http://medicinalplantgenomics.msu.edu/). Subsequently, amino acid sequences were deduced using Translate tool of ExPASY. (http://web.expasy.org/translate/) and their molecular features were analysed using EBI-Tools (http://www.ebi.ac.uk/Tools/emboss/).

The homology with known sequences was analysed using BLASTn and BLASTx tools of NCBI (https://blast.ncbi.nlm.nih.gov/Blast.cgi). Subcellular localization was predicted using Plant-mPloc prediction tool (http://www.csbio.sjtu.edu.cn/bioinf/plant-multi/)^110^. Evolutionary relationships of the sequences were compared using Maximum Likelihood method with thousand bootstrap values employing MEGA7 program. Genomic architecture of introns and exons was obtained using Gene Structure Display Server 2.0 (http://gsds.cbi.pku.edu.cn/)^111^.

### Isolation and cloning of full-length *C*. *roseus CWIN* CDS

Full-length *C*. *roseus CWIN* CDS was isolated *via* RT-PCR. Total RNA was isolated from leaf tissues of three month old *C*. *roseus* plants using RNeasy Plant Mini kit (Qiagen, Germany) following the manufacturer’s instructions. Subsequently, 3 µg of total RNA was subjected to DNase treatment and cDNA was synthesized using Transcriptor first strand cDNA synthesis kit (Roche, Germany). Full-length *C*. *roseus CWIN* CDS was amplified using gene specific primers (LP: 5′-GGATCCATGGCCAATTCTTACATTTGGTTCTTCT-3′; RP: 5′-GGTGACCCTCAATCTCACCATGATGAGAAATAAATTT-3′). Underlined bases contained *BamHI* and *SstI* sites respectively. The PCR-amplified CDS was cloned into pGEMT-Easy vector and validated by sequencing, restriction analysis and PCR. Next, the *C*. *roseus CWIN* CDS was cloned into the modified expression vector pCAMBIA2300 as *BamHI*-*SstI* insert, downstream to CaMV 35S Promoter.

### Transient overexpression of *C*. *roseus CWIN* CDS in *Nicotiana benthamiana*

Recombinant pCAMBIA2300 was transformed into *Agrobacterium tumefaciens* strain EHA105 *via* freeze thaw method^112^. Transformed clones were verified by PCR followed by agroinfiltration into *N*. *benthamiana* as previously reported^113^. Leaves infiltrated with recombinant agrobacterium carrying the vector pCAMBIA2301 were used as control. After four days, leaves were harvested, snap frozen in liquid nitrogen and stored at -80 °C until further analysis.

To study the effect of *CWIN* overexpression on other genes in *N*. *benthamiana*, total RNA was extracted from agroinfiltrated tissues. 6 µg of total RNA was used to synthesize cDNA and the gene-expression analysis was carried out *via* qRT-PCR. *PP2A*^114^ was used as an internal reference gene. The *N*. *benthamiana* genes analysed in this study were *4-coumarate:coenzyme a ligase* (*4-C*L; Nbv6.1trP58793), *Anthocyanidin Synthase* (*ANS*; Nbv6.1trP1132), *Cinnamoyl-CoA Reductase* (*CCR*; Nbv6.1trP67697), *Chalcone Synthase* (*CHS*; Nbv6.1trP67289), *Dihydroflavonol 4-Reductase* (*DFR*; Nbv6.1trP53078), *Flavanone 3-Hydroxylase* (*F3H*; Nbv6.1trP67389), *Shikimate o-Hydroxycinnamoyltransferase* (*HCT*; Nbv6.1trP21540), *Peroxidase 9* (*PRX*; Nbv6.1trP50659), *Catalase Isozyme 1* (*CAT*; Nbv6.1trP54093), *Superoxide Dismutase* (*SOD*; Nbv6.1trP67255), *Sucrose Synthase* (*SUSY;* Nbv6.1trP69162), *Sucrose Phosphate Synthase* isoforms (*SPS1; Nbv6*.*1trP64694*, *SPS2*, *Nbv6*.*1trP56089*), *Cell Wall Invertase* isoforms (*CWIN2; Nbv5*.*1tr6202472*, *CWIN3; Nbv5*.*1tr6228617*), *Phenylalanine Ammonia Lyase* isoforms (*PAL2*, Nbv6.1trP20094; *PAL3*, Nbv6.1trP49210 and *PAL4*, Nbv6.1trP56366), *Flavanone 3-hydroxylase* (*F3H*; Nbv6.1trP67389), *3-hydroxy-3-methylglutaryl-coenzyme-a-reductase 1* (*HMGR*; Nbv6.1trP54761), *Hydroxymethylglutaryl- synthase-like* (*HMGS*; Nbv6.1trP33093), *Probable 1-deoxy-d-xylulose-5-phosphate chloroplastic* (*DXS*; Nbv6.1trP16938), *1-deoxy-d-xylulose-5-phosphate reductoisomerase* (*DXR*, Nbv6.1trP48271), *Geraniol 8-hydroxylase-like* (*G8H*; Nbv6.1trP70636), *Cytochrome p450 cyp72a219-like* (*SLS*, Nbv6.1trP5153), *4-hydroxy-3-methylbut-2-en-1-yl diphosphate chloroplastic* (*HDS*; Nbv6.1trP30454), *Phytoene Synthase* (*PS*; Nbv6.1trP21364). The gene IDs have been obtained from Benth Genome (http://benthgenome.qut.edu.au). Primers used have been enlisted in Supplementary Table S1.

### CWIN activity assay

CWIN activity assay was performed as described previously^115^ with minor modifications. Briefly, the rapidly harvested leaf tissue was weighed, ground in liquid nitrogen followed by homogenization with 1 ml extraction buffer [all as mol m^−3^: Hepes-KOH (pH 8·0), 50; MgCl_2_, 5 Ethylenediaminotetraacetic acid (EDTA), 2; MnCl_2_, 1; CaCl_2_, 1; Benzamidine, 1; Dithiotreitol, 1; Phenyl-methylsulphonyl sulphonyl fluoride, 0·1] on ice. The homogenate was centrifuged at 13000 × g for 15 min at 4 °C and the pellet was resuspended in 500 µl extraction buffer. Total protein concentration in the extracts was determined using Bradford method^116^. The reaction mixture containing 10 µg of total protein, 200 mM sucrose and 50 mM sodium acetate buffer at pH 4.7 was incubated at 37 °C for 30 min. After incubation, reaction was alkalinized by adding 100 µl 1 M Tris-HCL, pH 8 and heated at 85 °C for 3 min. Two blanks were set up to measure acid hydrolysis of sucrose (contained no extract) and endogenous glucose levels (contained no sucrose). The amount of hexoses released was measured enzymatically using Sucrose, D-Fructose, D-Glucose assay kit (Megazyme, Ireland). Activity was expressed as micromoles of hexoses released per minute per milligram of total protein.

### Statistics

qRT-PCR data were analysed using REST^©^ and represented as box-and-whiskers plot, with central line indicating median of expression ratio with respect to control; box borders represent 95% confidence intervals and whiskers depict standard error margins. All other data are expressed as mean values and standard deviation of three independent experiments. Statistical significance was evaluated using t-test *via* GraphPad Prism 5 (GraphPad Software, La Jolla California USA, www.graphpad.com). All the graphs were plotted using GraphPad Prism 5 (GraphPad Software, La Jolla California USA, www.graphpad.com).

## Electronic supplementary material

Supplementary Information
