# Supplementary material for: Expression analysis of Cell wall invertase under abiotic stress conditions influencing specialized metabolism in Catharanthus roseus
Source: Sci Rep. 2018 Oct 10;8:15059. doi: 10.1038/s41598-018-33415-w (PMC6180051; doi:10.1038/s41598-018-33415-w)
Supplement: Supplementary file 1 — Supplementary Information [file 41598_2018_33415_MOESM1_ESM.pdf]

Expression analysis of *Cell wall invertase* under abiotic stress conditions influencing specialized metabolism in *Catharanthus roseus*

Nishanth M J[1], Sheshadri S A[1], Sudarshan Singh Rathore[2], Srinidhi S[1], Bindu Simon[1]*

[1] Phytoengineering Lab, School of Chemical and Biotechnology, SASTRA Deemed to be University
[2]Actinomycetes Bioprospecting Lab, School of Chemical and Biotechnology, SASTRA Deemed to be University
Correspondence: bindusimon@scbt.sastra.edu
Phone: +91-4362-264101

Supplementary Figure-1a: Chromatograms of *Catharanthus roseus* samples recorded at 210 nm

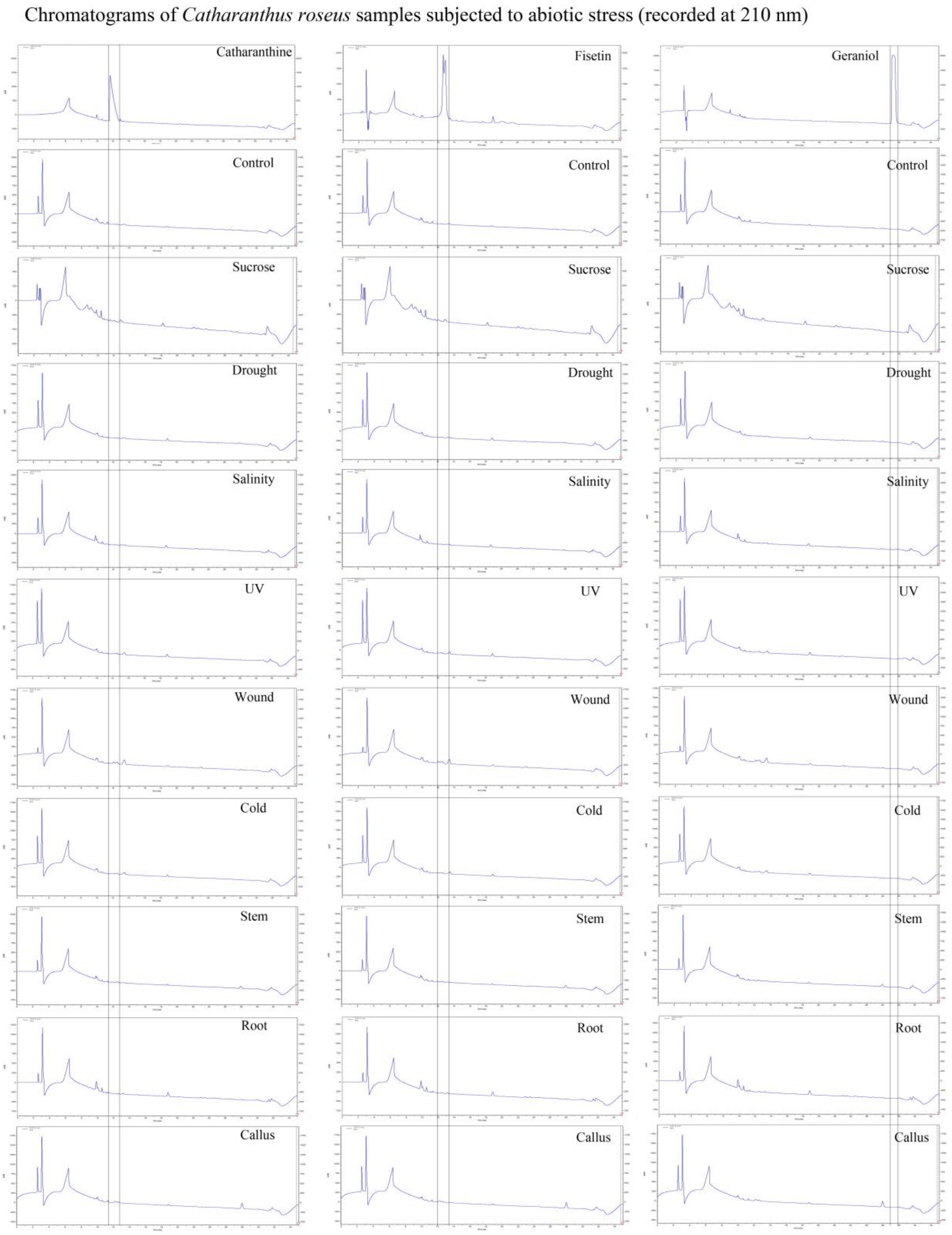

Supplementary Figure-1b: Chromatograms of *Catharanthus roseus* samples recorded at 280 nm

Chromatograms of *Catharanthus roseus* samples subjected to abiotic stress (recorded at 280 nm)

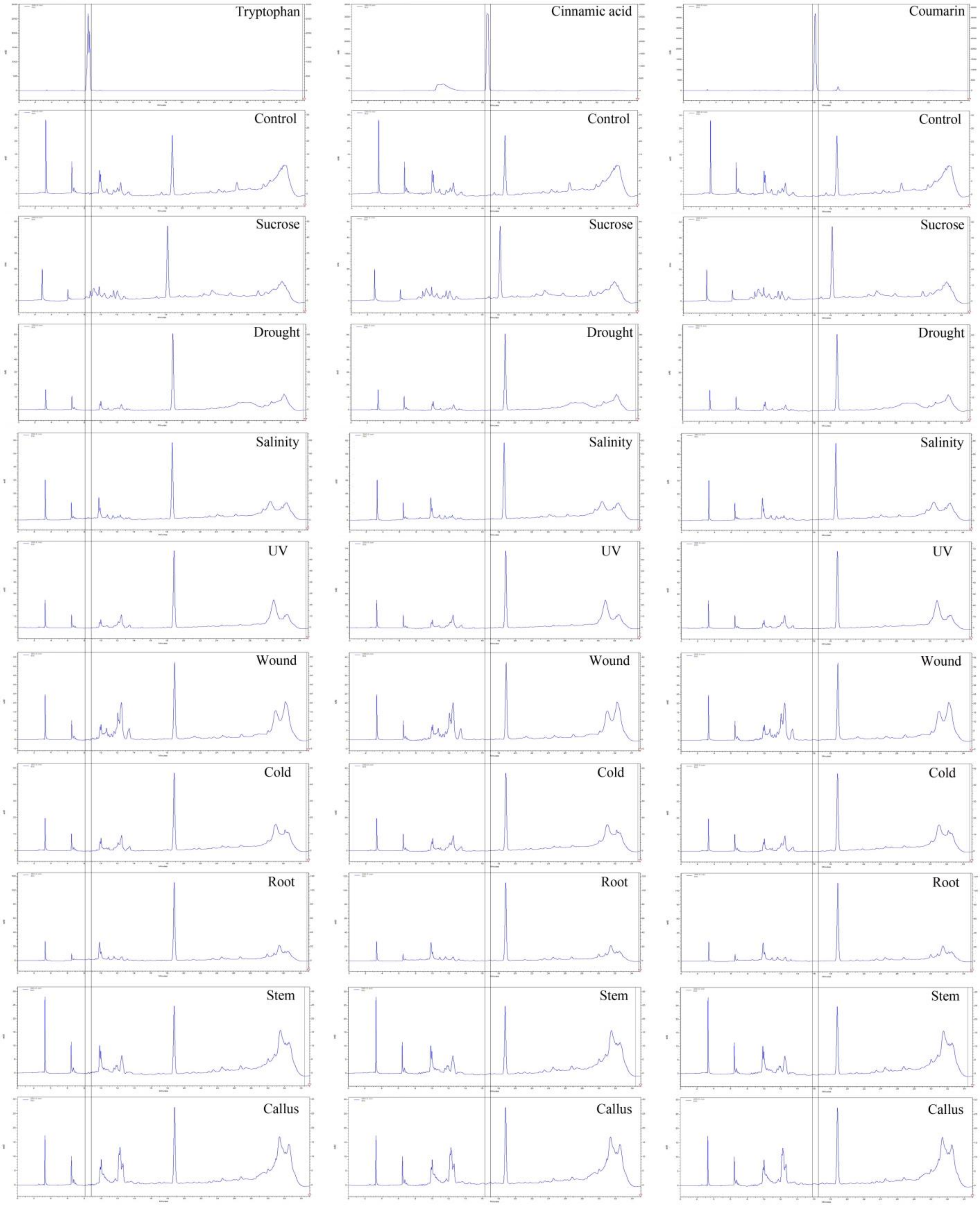

Supplementary Figure-1c: Chromatograms of *Catharanthus roseus* samples recorded at 269 nm

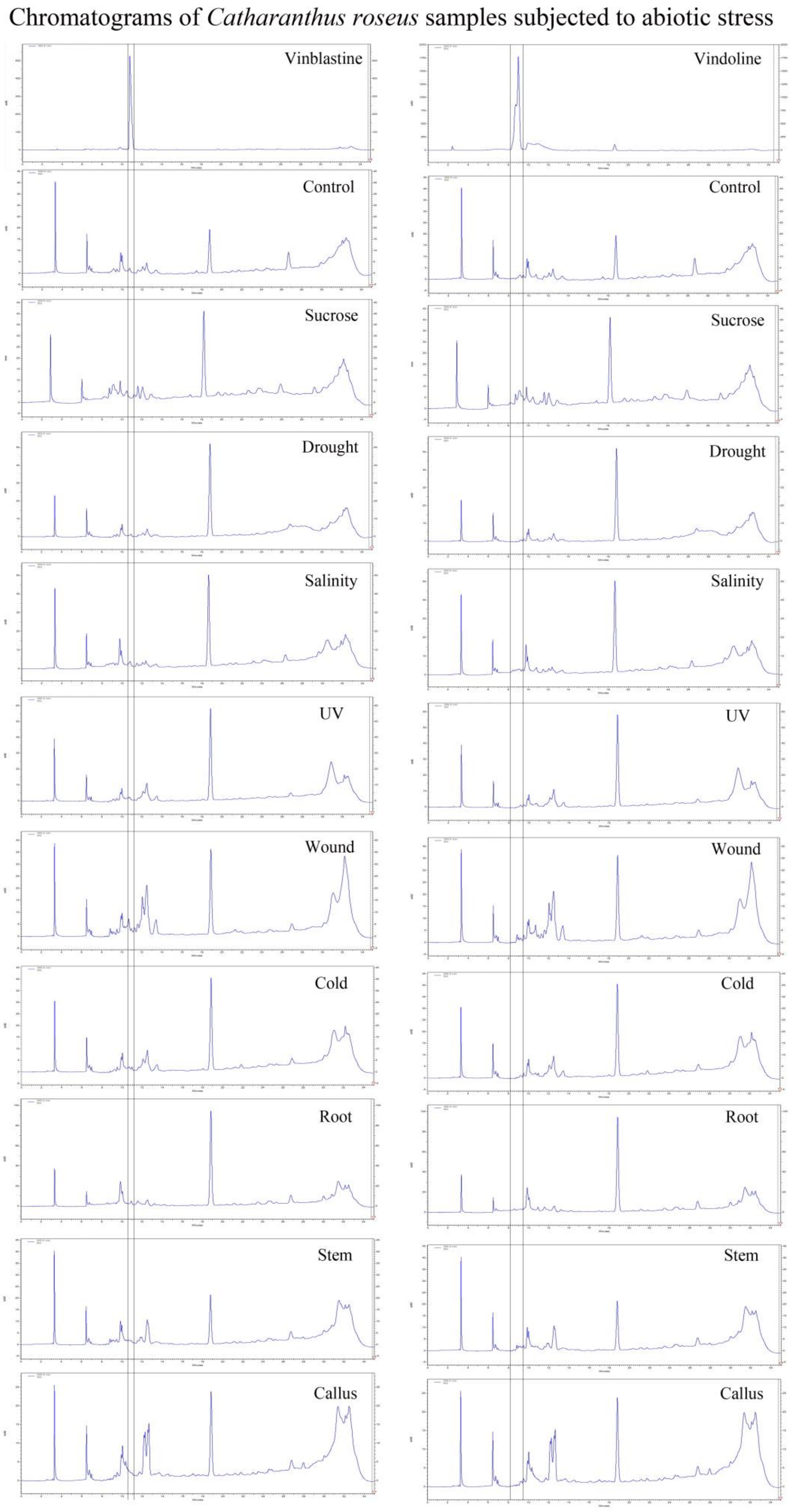

Supplementary Figure-2: Chromatograms of *Nicotiana benthamiana* samples recorded at 210 nm, 250nm and 280nm

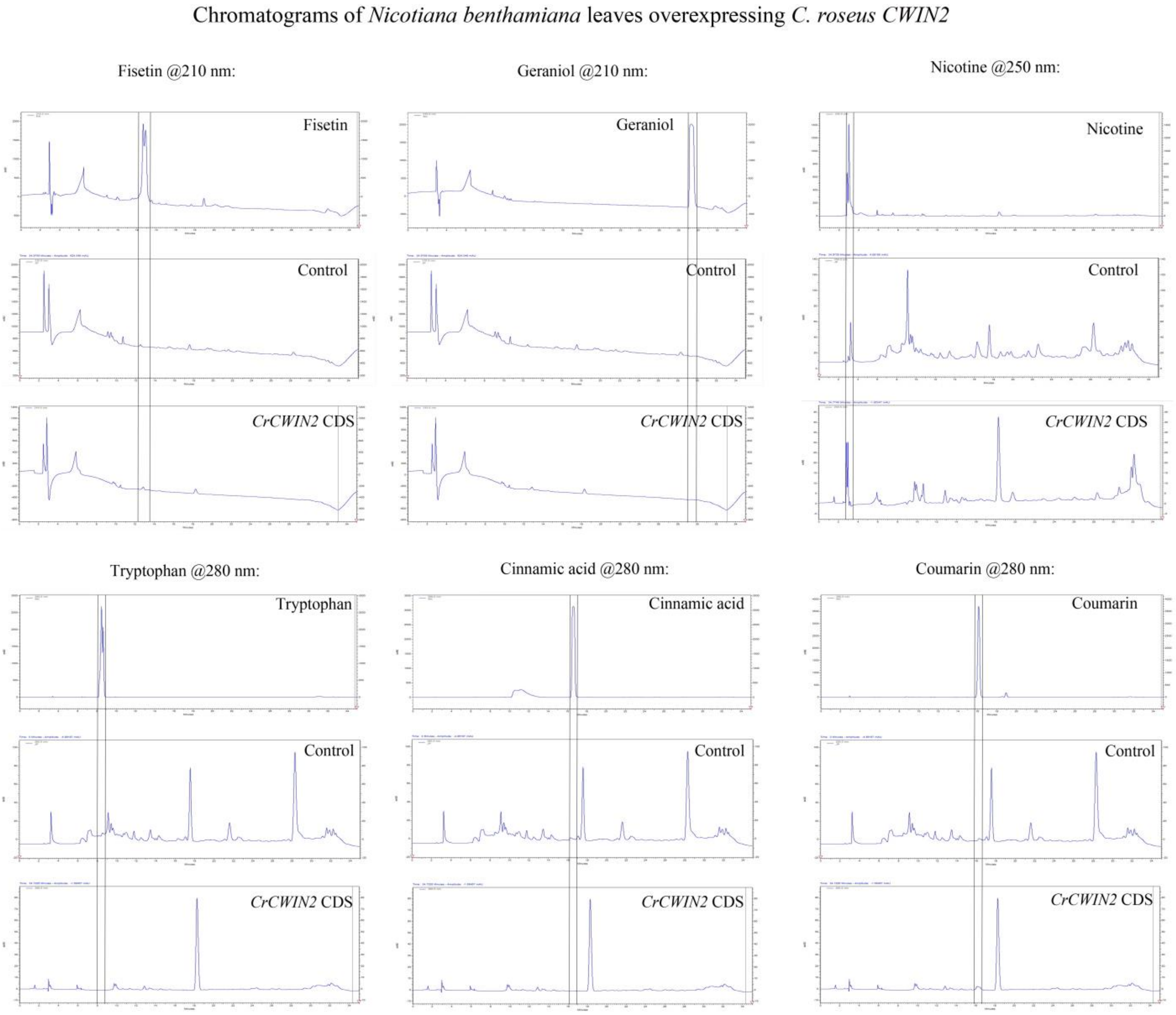

Supplementary table S1: Genes and primer details

C. roseus

S.N	Gene name and symbol	Sequence ID (based on MPGR database)	Scaffold	Forward primer/ Reverse primer
1	*Cell Wall Invertase 1 (CrCWIN1)*	CRO_T000083	cro_scaffold_3070386	TCCATAAGGTCAGTCTTGATGC/ TCCAACCTTAGTCCTTTCCAG
2	*Cell Wall Invertase 2 (CrCWIN2)*	CRO_T031716	cro_scaffold_3060381	CAATTCAACTTCATTTAGAGATCCA/ TGCTCTTCCTTTTCTATTGATTTTG
3	*Cell Wall Invertase 3 (CrCWIN3)*	CRO_T020329	cro_scaffold_3065222	ATTGTGGGGATGGGTAACTG/ CTGCTTTGTCGAGCCATACA
4	*Sucrose Phosphate Synthase(SPS)*	CRO_T025530	cro_scaffold_3062068	GGGTCCACAGAAAAAGGTGA/ CTTCTCCGCAGCATCAAATA
5	*Sucrose Synthase (SUSY)*	CRO_T016709	cro_scaffold_3056582	GCTGCTACACCATCTCTCAAG/ TGGATTTCTCCATTTCATCCA
6	*Geraniol-10-Hydroxylase (G10H)*	CRO_T015220	cro_scaffold_3061275	TTATTCGGATTCTGCCAAGG/ ACTTCCCCAAAGTGAATCGTC
7	*Catalase (CAT)*	CRO_T019281	cro_scaffold_3065736	TTTCTTCGAGCTCCTGGTGT/ GTGGGACTTGGGGTTAGGTT
8	*Super Oxide Dismutase (SOD)*	CRO_T011284	cro_scaffold_3007309	AAACATGGAATTGGCAAGGA/ GCGGCTCTTTTCTAGTTTGG
9	*Peroxidase 1 (PRX1)*	CRO_T002898	cro_scaffold_3059391	CTTGCTTCGCCTTCATTTTC/ GATCCTGAAAGCCTGCTGAC
10	*1-deoxyxylulose 5-phosphate synthase (DXS)*	CRO_T025084	cro_scaffold_2986453	TCGCTGCAGAACTTAGAGCA/ GCCAACATCCCAAATGATTC
11	*deacetylvindoline 4-O-acetyltransferase (DAT)*	CRO_T020280	cro_scaffold_3060125	TGACGATGGAATTGAGTTCG/ CCGTTTGGGAAGGACTAGGT
12	*Chalcone and stilbene synthase family protein (CHS)*	CRO_T014717	cro_scaffold_2987430	CGGCACAGTTCTTCGATTG/ GTCTCCGAACAAGGCTTGAC
13	*Phenylalanine Ammonia Lyase(PAL)*	CRO_T013529	cro_scaffold_3063391	GATTTGAGGCATTTGGAGGA/ CGAAAACATATTCGCGGTCT
14	*Secologanin synthase(SLS)*	CRO_T024556	cro_scaffold_3063455	CTTTGAGGGTGCAAAATGGT/ TGGGATCCTTGTTTTTCAGC
15	*AP2-domain DNA-binding protein (ORCA3)*	CRO_T030273	cro_scaffold_3061731	GGGATCCGAAAAAGAAAGGA/ AGCTCCACGCATATTAAACG
16	*Tryptophan deCarboxylase (TDC)*	CRO_T006098	cro_scaffold_3045674	CGCCTGTATATGTCCCGAGT/ GTTGCGATTTGCCAATTTTT
17	*Strictosidine Synthase(STR)*	CRO_T006099	cro_scaffold_3045674	ACCATTGTGTGGGAGGACAT/ CCATTTGAATGGCACTCCTT
18	*Cinnamate-4-Hydroxylase (C4H)*	CRO_T000894	cro_scaffold_2986973	GGAAACTGGCTTCAAGTTGG/ CCCCTGAGTGTGCAAAACTT
19	*senescence-associated gene (SAG)*	CRO_T020296	cro_scaffold_3065953	ACCATGGATTGGAGGATCAA/ TGTCACAATCCACTAGTTCTTGC
20	*Sand protein (SAND)*	CRO_T002730	cro_scaffold_3024641	TTGACCCTGCTTCTCGTTCT/ GCAAGCTGCTGATAGGTGAG

N. benthamiana

S.N	Gene name and symbol	Sequence ID*	Forward primer/ Reverse primer
1	*Protein phosphatase 2A (NbPP2A)*	Nbv6.1trP16930	GACCCTGATGTTGATGTTCGCT/ GAGGGATTTGAAGAGAGATTTC
2	*Cell Wall Invertase 2 (NbCWIN2)*	Nbv5.1tr6202472	ATTGTTTGCATGAAGCATCAGA/ GCAGCTTCAAATTGCCGAAT
3	*Cell Wall Invertase 3 (NbCWIN3)*	Nbv5.1tr6228617	TTCAGTTCTAAAAGGAGAGATGGA/ AGATGAGCTAGTAGATTGCAAATG
4	*Phenylalanine Ammonia Lyase 2 (NbPAL2)*	Nbv6.1trP20094	GGTCATGAACAACAAGACCCTTT/ GGCCACCTGACCTATCGTT
5	*Phenylalanine Ammonia Lyase 3 (NbPAL3)*	Nbv6.1trP49210	CTCCAAAATATACCTTCAATTATTCAC/ TCCACTTTAACGCAAAAATCC
6	*Phenylalanine Ammonia Lyase 4 (NbPAL4)*	Nbv6.1trP56366	CTTCGTTTCTCATAGCTAACAACA/ TGTTGATGACCCTTAATGCAA
7	*Peroxidase 9 (NbPRX)*	Nbv6.1trP50659	GCCAGATGCAACACTTGAGA/ CAAAAGTCCTTTGCCCCATA
8	*Superoxide Dismutase (NbSOD)*	Nbv6.1trP67255	GAGCAAACAGGTCGAAAAGC/ CCCTCGTCAAATCACCAAAT

9	*4-coumarate:coenzyme a ligase (Nb4CL)*	Nbv6.1trP58793	TGGTTACACACTGGCGACAT/ GGACAACAGCAGCATCAGAA
10	*Cinnamoyl- reductase (NbCCR)*	Nbv6.1trP67697	CATTTGAGGGAGCTTGAAGG/ CGAAGTGAACACCACACGTC
11	*Anthocyanidin synthase (NbANS)*	Nbv6.1trP1132	ATGCCAAGCAGATCAGGAAC/ AGGCACCATATTGTGGAGGA
12	*Chalcone synthase (NbCHS)*	Nbv6.1trP67289	CCAGCCCAAATCCAAGATTA/ CGGTGATCTCTGAGCAAACA
13	*Dihydroflavonol 4-reductase (NbDFR)*	Nbv6.1trP53078	ATTGGAATTGCCAAAAGCTG/ CATTCCCCTGACTGTTGGTT
14	*Flavanone 3-hydroxylase (NbF3H)*	Nbv6.1trP67389	AAAGTGTCCACAGCCTGACC/ TTCGAGTTCACCACTGCTTG
15	*Shikimate o-hydroxycinnamoyltransferase (NbHCT)*	Nbv6.1trP21540	CCAAGTCCAAGGAAGATGGA/ GATATCACCAGCCACTGCAA
16	*Sucrose Synthase (NbSUSY)*	Nbv6.1trP69162	GCAGTTCCATTGGCTATTGAG/ TTCAAATTTGCAATCCTCTTGA
17	*Sucrose Phosphate Synthase 1 (NbSPS1)*	Nbv6.1trP64694	GCTCTTTTGAAGCTGGTTGC/ CATCATCATCGGTTCTCAGC
18	*Sucrose Phosphate Synthase 2 (NbSPS2)*	Nbv6.1trP56089	GGCAAAGAAGGTAGATGATATGAGA/ GAGCGGAACAACTTGCATTC
19	*3-hydroxy-3-methylglutaryl-coenzyme a reductase 1 (HMGR)*	Nbv6.1trP54761	CTGTTCCTCCACCACCTGTT/ TAATCCTCTGCAACGCCTCT
20	*Hydroxymethylglutaryl- synthase-like (NbHMGS)*	Nbv6.1trP33093	CTCTTCATGGGATGGACGTT/ AGCCCTGATCTTGCTTTCAA
21	*1-deoxy-d-xylulose-5-phosphate synthase (DXS)*	Nbv6.1trP16938	TGAATATGCTCGTGGGATGA/ TGGCAACTCCGTGATACTTG
22	*1-deoxy-d-xylulose-5-phosphate reductoisomerase (NbDXR)*	Nbv6.1trP48271	AGGGACTTGCCAGTTGAGAA/ GTATGATGGACTGGGGATGG
23	*Geraniol 8-hydroxylase-like (NbG10H)*	Nbv6.1trP70636	CGACATTAGCCCAGAAGAGC/ AGTTTGCCTCTGCCAATCAC
24	*Cytochrome p450 cyp72a219-like/ Secologanin synthase (NbSLS)*	Nbv6.1trP5153	TGATCTTGCACGAGGTTCTG/ TTCTCCCCATTGGTCTGTGT
25	*4-hydroxy-3-methylbut-2-en-1-yl diphosphate synthase (NbHDS)*	Nbv6.1trP30454	GCTCCTCCTATTGCACTTCG/ AAAGGCTCCCGTGGTTAGTT
26	*Phytoene synthase (NbPS)*	Nbv6.1trP21364	AGAAGGGCTATTTGGGCAAT/ CAGTATCGGACAAAGCAGCA
27	*Senescence-associated gene (SAG)*	Nbv6.1trP4953	GGAGGACTCTTGGACACTGC/ CTGCCTGCAATAGAGCCTTC

* Sequence ID's are based on the *Nicotiana benthamiana* genome and transcriptome database (http://benthgenome.qut.edu.au/)
